# Transgenic Expression of the Formin Protein Fhod3 Selectively in the Embryonic Heart: Role of Actin-Binding Activity of Fhod3 and Its Sarcomeric Localization during Myofibrillogenesis

**DOI:** 10.1371/journal.pone.0148472

**Published:** 2016-02-05

**Authors:** Noriko Fujimoto, Meikun Kan-o, Tomoki Ushijima, Yohko Kage, Ryuji Tominaga, Hideki Sumimoto, Ryu Takeya

**Affiliations:** 1 Departments of Biochemistry, Kyushu University Graduate School of Medical Sciences, 3-1-1 Maidashi, Higashi-ku, Fukuoka 812–8582, Japan; 2 Departments of Pharmacology, Faculty of Medicine, University of Miyazaki, 5200 Kihara, Kiyotake, Miyazaki 889–1692, Japan; 3 Departments of Cardiovascular Surgery, Kyushu University Graduate School of Medical Sciences, 3-1-1 Maidashi, Higashi-ku, Fukuoka 812–8582, Japan; University of Maryland, UNITED STATES

## Abstract

Fhod3 is a cardiac member of the formin family proteins that play pivotal roles in actin filament assembly in various cellular contexts. The targeted deletion of mouse *Fhod3* gene leads to defects in cardiogenesis, particularly during myofibrillogenesis, followed by lethality at embryonic day (E) 11.5. However, it remains largely unknown how Fhod3 functions during myofibrillogenesis. In this study, to assess the mechanism whereby Fhod3 regulates myofibrillogenesis during embryonic cardiogenesis, we generated transgenic mice expressing Fhod3 selectively in embryonic cardiomyocytes under the control of the β-myosin heavy chain (MHC) promoter. Mice expressing wild-type Fhod3 in embryonic cardiomyocytes survive to adulthood and are fertile, whereas those expressing Fhod3 (I1127A) defective in binding to actin die by E11.5 with cardiac defects. This cardiac phenotype of the Fhod3 mutant embryos is almost identical to that observed in Fhod3 null embryos, suggesting that the actin-binding activity of Fhod3 is crucial for embryonic cardiogenesis. On the other hand, the β-MHC promoter-driven expression of wild-type Fhod3 sufficiently rescues cardiac defects of Fhod3-null embryos, indicating that the Fhod3 protein expressed in a transgenic manner can function properly to achieve myofibril maturation in embryonic cardiomyocytes. Using the transgenic mice, we further examined detailed localization of Fhod3 during myofibrillogenesis *in situ* and found that Fhod3 localizes to the specific central region of nascent sarcomeres prior to massive rearrangement of actin filaments and remains there throughout myofibrillogenesis. Taken together, the present findings suggest that, during embryonic cardiogenesis, Fhod3 functions as the essential reorganizer of actin filaments at the central region of maturating sarcomeres via the actin-binding activity of the FH2 domain.

## Introduction

Myofibrils, a contractile structure in striated muscles, are composed of functional repeating units called sarcomeres, which are highly organized arrays of thin actin filaments and myosin-based thick filaments [1]. Actin filaments in the sarcomere are anchored to the boundary of the sarcomere (the Z line) via interactions with the barbed end capping protein CapZ, and their pointed ends are directed toward the midline of the sarcomere (the M line). During myofibrillogenesis, actin filaments are dynamically organized into highly ordered mature structures from an irregularly-oriented state with a striking increase of their content [2,3]. Although mechanisms for the regulation of actin dynamics during myofibrillogenesis have remained largely unknown, various actin-binding proteins, including tropomodulin (Tmod), troponin T, and α-tropomyosin are known to contribute to this process [4–6]. Fhod3, a member of the formin family proteins, is another probable candidate for a key regulator of actin dynamics during myofibrillogenesis.

Formin family proteins, structurally characterized by the presence of the formin-homology domains 1 and 2 (FH1 and FH2), play pivotal roles in remodeling the actin and microtubule cytoskeletons [7–9]. The FH2 domain directly binds to G- and F-actin and mediates actin filament nucleation and polymerization, which are accelerated by the FH1-mediated recruitment of the profilin–actin complexes [10]. Through cooperation of the FH1 and FH2 domains, formins contribute to various biological functions via regulation of actin dynamics. Recent studies using genetically engineered animals revealed that various formins play critical roles in morphogenesis and organogenesis during development [11,12].

Fhod3, a cardiac member of formins, plays an essential role in the regulation of the actin assembly in cardiac myofibrils. We and another group have previously shown that RNA interference-mediated depletion of Fhod3 in cultured cardiomyocytes disrupts sarcomere organization [13,14]. In addition, we have recently shown that genetic depletion of Fhod3 in mice confers embryonic lethality with defects in cardiogenesis [15]. In Fhod3 null embryos, premyofibrils are formed once but failed to maturate, suggesting that Fhod3 plays an essential role in myofibrillogenesis, particularly in the maturation of myofibrils. Since this maturation process requires extensive reorganization of actin filaments, *i*.*e*., a massive increase in F-actin content and precise alignment of actin filaments, Fhod3 is expected to function as a key regulator for this process of actin organization. However, it remains unknown how Fhod3 functions during this process.

Whether Fhod3 directly acts on actin filament during cardiogenesis is an important issue to be elucidated. Although a direct binding to the barbed end of the actin filament appears to be a common feature of the FH2 domains, some formins do not appear to necessitate their FH2 domains for their physiological functions [16,17]. In this context, we cannot exclude the possibility that the FH2 domain of Fhod3 is dispensable for cardiogenesis. In our previous study, the α-MHC promoter-driven expression of a mutant Fhod3, defective in binding to actin, resulted in postnatal lethality rather than embryonic lethality, indicating that the actin binding activity of the FH2 domain is necessary at least after birth, *i*.*e*., after completion of myofibrillogenesis [15]. It thus remains to be elucidated whether the actin binding activity via the FH2 domain is required for myofibrillogenesis during cardiac development.

On the other hand, defining the precise localization of Fhod3 in developing myofibrils is also expected to provide insight into the understanding of the mechanism for Fhod3 function. In our previous study, we detected only weak signals at the central region of sarcomeres at E11.5 and E13.5, but no significant signals at the earlier stage (*i*.*e*., before E11.5) [15]. This was probably due to the relatively low sensitivity of Fhod3 antibodies, since the Fhod3 expression in the heart at this stage was clearly shown by the *lacZ* staining [15]. Consistent with this, Iskratsch *et al*. have detected only faint signals of Fhod3 diffusely distributed at E9.5 [18]. However, given that Fhod3 plays an essential role in sarcomeric organization at this stage [15], it is reasonably expected that Fhod3 localizes and functions at the specific site in the maturating sarcomere, although we cannot detect it using the currently available Fhod3 antibodies. In this context, overexpression is a useful approach to identifying Fhod3 localization; we have previously shown that Fhod3 protein expressed in a transgenic manner distributes in the same pattern as the endogenous protein, but more intensely [19].

Here we have generated transgenic mice expressing Fhod3 selectively in embryonic cardiomyocytes using the β-myosin heavy chain (MHC) promoter. Since the β-MHC promoter-driven expression of wild-type Fhod3 sufficiently rescues cardiac defects of Fhod3-null embryos, the Fhod3 protein expressed is supposed to function properly to achieve myofibril maturation during embryonic cardiogenesis. Using the transgenic mice, we have successfully observed the localization of Fhod3 during myofibrillogenesis. In addition, mice expressing a mutant Fhod3 defective in binding to actin die by E11.5 with cardiac defects; the phenotype of which is almost identical to that observed in Fhod3 null embryos, suggesting that the actin-binding activity is crucial for Fhod3 function during embryonic cardiogenesis. Taken together, our present findings suggest that, during embryonic cardiogenesis, Fhod3 functions critically at the central region of maturating sarcomeres via the actin-binding activity of the FH2 domain.

## Materials and Methods

### Ethics Statement

All procedures using mice were performed in strict accordance with the guidelines for Proper Conduct of Animal Experiments (Science Council of Japan). The experimental protocol was approved by the Animal Care and Use Committee of Kyushu University (Permit Number: A26- 102). All efforts were made to minimize the number of animals used and their suffering.

### Mice

Transgenic mice expressing wild-type Fhod3 that contained all the 28 exons [19] or a mutant Fhod3 carrying the I1127A substitution [13] under the control of the β-myosin heavy chain (β-MHC) promoter, a generous gift from Dr. Jeffery Robbins (Cincinnati Children’s Hospital Medical Center) [20], were generated on a C57BL/6 background. Four different founder mice expressing a mutant Fhod3-I1127A were obtained, but only three mice surviving to reproductive age (#1, female; #2, female; #4, male) were crossed with C57BL/6 mice to generate F1 progeny. For analysis of embryos, the transgenic male mouse #4 were crossed with C57BL/6 female mice and pregnant females were used. We obtained only one transgenic line expressing wild-type Fhod3 from over 200 injections, which was used for immunofluorescent microscopic analysis and for rescue experiments with the Fhod3 knockout mouse. All mice were kept in a specific pathogen-free animal facility at Kyushu University.

### Antibodies

Rabbit polyclonal antibodies specific for Fhod3 (anti-Fhod3-(650–802) and anti-Fhod3-C20) were prepared as previously described [21]. The mouse monoclonal antibody against α-actinin (clone EA-53) was purchased from Sigma-Aldrich; the rabbit polyclonal antibodies against Tmod1 from ProteinTech Group; the goat polyclonal antibodies against myomesin-1 (C-16) from Santa Cruz; the mouse monoclonal antibody against β-myosin heavy chain (NOQ7.5.4D) [22] from Abcam; Alexa Fluor 488-conjugated F(ab’)_2_ fragment of anti-mouse IgG and Alexa Fluor 555-conjugated F(ab’)_2_ fragment of anti-rabbit IgG from Cell Signaling Technology; and Alexa Fluor 594 phalloidin and Alexa Fluor 647 phalloidin from Life Technologies.

### Histological analysis

Histological analysis was performed as previously described [15,19]. Briefly, timed pregnant mice were sacrificed via cervical dislocation and embryos were dissected from the uterus. Dissected embryos were fixed by immersion in a solution containing 3.7% formaldehyde in phosphate-buffered saline (PBS; 137 mM NaCl, 2.68 mM KCl, 8.1 mM Na_2_HPO_4_, and 1.47 mM KH_2_PO_4_, pH 7.4) at 4°C. In the case of embryos at E18.5, the heart was removed from mice under hypothermal anesthesia and then fixed. Fixed embryos or hearts were dehydrated in ethanol, embedded in paraffin, sectioned, and stained with hematoxylin and eosin.

### Immunofluorescence staining

Immunofluorescence staining was performed as previously described [15,19]. Briefly, timed pregnant mice were sacrificed via cervical dislocation and the uterus was removed. The whole body of embryos at E9.5–11.5 or the hearts dissected under hypothermal anesthesia from embryos at E13.5–17.5 were fixed by immersion in 3.7% formaldehyde for 12 h at 4°C. The fixed whole embryo or heart was washed in PBS, subjected to osmotic dehydration overnight at 4°C in 30% sucrose, and embedded in OCT compound (Sakura Finetek). The blocks were frozen and cut into 5 μm sections using a cryostat (HM550; Thermo Scientific). Then sections were washed with PBS containing 0.1% Triton X-100, and blocked with a blocking buffer (Blocking One Histo; Nacalai tesque) for 5 min at room temperature. Sections were labeled overnight at 4°C with primary antibodies diluted in a dilution buffer (PBS containing 3% bovine serum albumin, 2% goat serum, and 0.1% Triton X-100), and then labeled for 2 h at room temperature with a fluorescein-conjugated secondary antibody mixture in the same buffer. For Fhod3 staining, anti-Fhod3-(650–802) antibodies were used. Images were taken with LSM700 or LSM5 PASCAL confocal scanning laser microscope (Carl Zeiss MicroImaging).

### Immunoblot analysis

Immunoblot analysis was performed as previously described [15,19]. Briefly, the hearts of mice were homogenized and sonicated at 4°C in a lysis buffer (10% glycerol, 135 mM NaCl, 5 mM EDTA, and 20 mM Hepes, pH 7.4) containing Protease inhibitor cocktail (Sigma-Aldrich). In the case of S1 Fig, the hearts of mice were snap-frozen, crushed using SK-Mill (SK-100; FUNAKOSHI), and dissolved in a buffer composed of 9 M Urea, 2% SDS, 2% Triton X-100, 1% dithiothreitol, and 10 mM Tris-HCl, pH6.8. The lysates were applied to SDS-PAGE and transferred to a polyvinylidene difluoride membrane (Millipore). The membrane was probed with the anti-Fhod3-C20 antibodies, followed by development using ECL-plus (GE Healthcare) for visualization of the antibodies.

### Transfection and immunoprecipitation

The cDNA fragment for Fhod3-ΔN, lacking the N-terminal 930 amino acids, was constructed by PCR using the cDNA encoding mouse Fhod3 of 1,586 amino acids, which contains all the 28 exons, as previously described [19]. The DNA fragments were ligated to pEGFP-C1 (Clontech) or pEF-BOS for expression in HEK293 cells as an N-terminally green fluorescent protein (GFP)–tagged protein or FLAG–tagged protein, respectively. All the constructs were sequenced for confirmation of their identities. Immunoprecipitation analysis was performed as previously described [23]. Briefly, HEK293 cells were transfected with indicated plasmids using X-tremeGENE 9 (Roche) and cultured for 24 hours in DMEM supplemented with 10% FCS. The cells were lysed at 4°C with a lysis buffer (10% glycerol, 135 mM NaCl, 5 mM EDTA, and 20 mM Hepes, pH 7.4) containing 0.1% Triton X-100. The lysates were precipitated with an anti-HA antibody (3F10, Roche) in the presence of protein G-Sepharose. After washing three times with the lysis buffer containing 0.1% Triton X-100, the precipitants were applied to SDS-PAGE and transferred to a polyvinylidene difluoride membrane (Millipore). The membrane was probed with anti-Flag polyclonal antibodies, anti-HA monocolonal antibody (16B12), or the anti-Fhod3-C-20 antibodies, followed by development using ECL-prime (GE Healthcare) for visualization of the antibodies.

## Results

### Transgenic expression of an actin binding-defective Fhod3 in the embryonic heart

**Fig 1:**
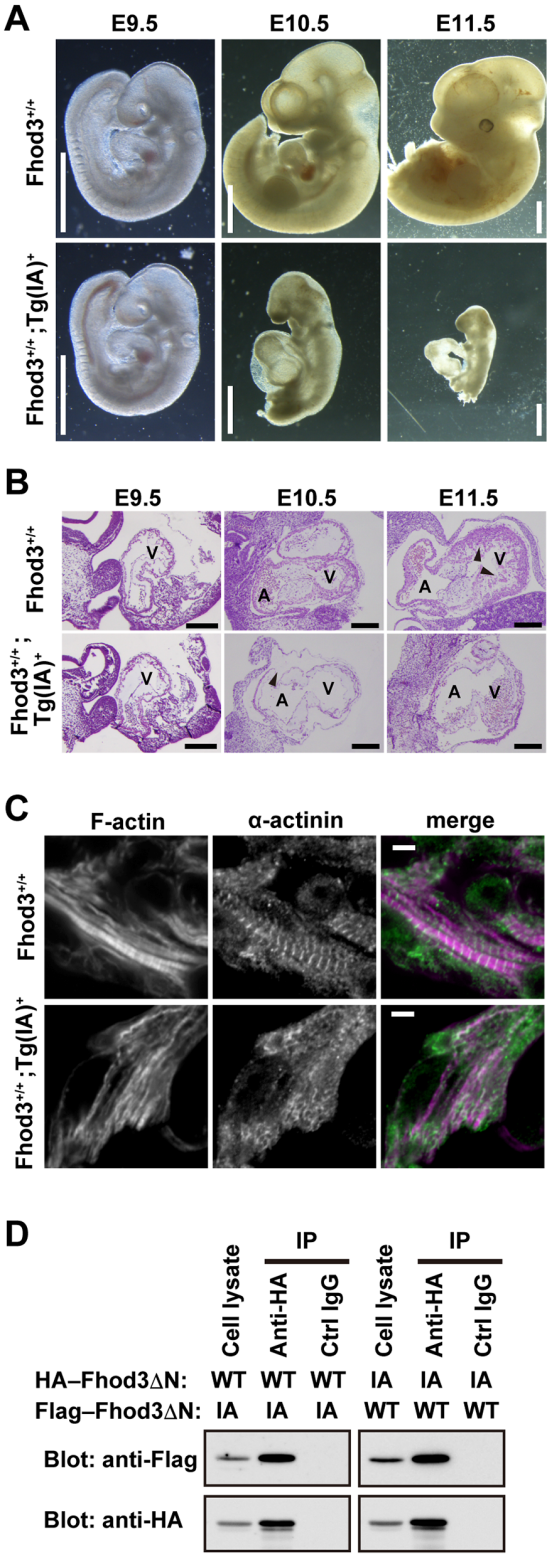
Effect of transgenic expression of Fhod3-I1127A in the embryonic heart. (A) Whole-mount analysis of nontransgenic wild-type (*Fhod3*^**+/+**^) and transgenic *Fhod3*^**Tg(β-MHC-Fhod3IA)**^ (*Fhod3*^**+/+**^;*Tg(IA)*^**+**^) embryos at E9.5, E10.5, and E11.5. The caudal portion of some embryos was resected for genotyping experiments. Scale bars, 1 mm. (B) Histological analysis of nontransgenic wild-type (*Fhod3*^**+/+**^) and transgenic (*Fhod3*^**+/+**^;*Tg(IA)*^**+**^) embryos at E9.5, E10.5, and E11.5. Longitudinal sections of hearts were stained with hematoxylin and eosin. A, atrium; V, ventricle. Arrowheads indicate ventricular trabeculae. Scale bars, 200 μm. (C) Confocal fluorescence micrographs of cardiac myofibrils of nontransgenic wild-type (*Fhod3*^**+/+**^) and transgenic (*Fhod3*^**+/+**^;*Tg(IA)*^**+**^) embryos at E9.5. Sections of embryonic hearts were subjected to immunofluorescent staining for α-actinin (green) and phalloidin staining for F-actin (magenta). Scale bars, 5 μm. (D) Proteins in lysates of HEK293 cells expressing indicated proteins (Cell lysate) were immunoprecipitated (IP) with the anti-HA or control IgG, and then analyzed by immunoblot with the indicated antibodies.

To investigate the role of the actin binding activity of the FH2 domain in Fhod3 during cardiogenesis, we generated transgenic mice expressing a mutant Fhod3 carrying the I1127A substitution under the control of the β-MHC promoter (*Fhod3*^Tg(β-MHC-Fhod3IA)^). It is known that the mutant protein, defective in binding to actin, fails to induce actin assembly in HeLa cells and to promote sarcomere organization in cultured cardiomyocytes [13]. Among four founder *Fhod3*^Tg(β-MHC-Fhod3IA)^ mice obtained, three mice surviving to the reproductive age were crossed with C57BL/6 mice to generate F1 progeny. However, the crosses of the founder mice with C57BL/6 mice did not produce any Tg(+) mice in their litters (Table 1), indicative of embryonic death. To determine the stage of embryonic lethality, timed matings were performed using the male founder *Fhod3*^Tg(β-MHC-Fhod3IA#4)^. Genotype analysis of embryos revealed that Tg(+) embryos were present until E12.5, but not beyond that point (Table 2). Morphological analysis of embryos revealed that *Fhod3*^Tg(β-MHC-Fhod3IA)^ embryos (*Fhod3*^**+/+**^;*Tg(IA)*^**+**^) at E9.5 were comparable to nontransgenic wild-type embryos (*Fhod3*^**+/+**^) in size and gross morphology, appearing remarkably normal (Fig 1A), although the exogenous Fhod3 protein was already abundantly expressed at this time point (S1 Fig). The growth of *Fhod3*^Tg(β-MHC-Fhod3IA)^ embryos was stunted thereafter and died around E11.5 with pericardial effusion. As demonstrated by histological analysis of the heart in Fig 1B, the increment of myocardial mass of the ventricular wall with trabeculation, which is observed in nontransgenic embryos, was impaired in *Fhod3*^Tg(β-MHC-Fhod3IA)^ embryos. These phenotypes of *Fhod3*^Tg(β-MHC-Fhod3IA)^ embryos were strikingly similar to those of Fhod3 null embryos [15]. We next examined the myofibril structure in the heart of *Fhod3*^Tg(β-MHC-Fhod3IA)^ embryos and non-Tg embryos by immunofluorescence staining for sarcomeric α-actinin. As shown in Fig 1C, maturation defects of sarcomeres, such as α-actinin aggregates and continuous F-actin, were observed in the *Fhod3*^Tg(β-MHC-Fhod3IA)^ mice heart at E9.5. Thus *Fhod3*^Tg(β-MHC-Fhod3IA)^ embryos were grossly stunted and malformed with aborted development of the myocardium at mid-gestation. Taken together with the finding that the mutant Fhod3IA protein can interact with the wild-type Fhod3 protein (Fig 1D), the exogenous Fhod3IA protein is proposed to form a non-functional heterodimer with the endogenous wild-type Fhod3 protein, leading to a dominant negative effect. These findings suggest that the actin binding-activity of Fhod3 is crucial for myofibrillogenesis during cardiac development.

**Table 1:** Genotypes of offspring from mating of β-MHC-Fhod3-IA × B6.

mating (female × male)	non-Tg	Tg(IA)
β-MHC-Fhod3-IA#1 × B6	21	0
β-MHC-Fhod3-IA#2 × B6	27	0
B6 × β-MHC-Fhod3-IA#4	66	0

**Table 2:** Genotypes of embryos from mating of β-MHC-Fhod3-IA#4 × B6.

Stage	non-Tg	Tg(IA)
E9.5	12	7
E10.5	5	3
E11.5	12	4
E12.5	7	1
E16.5	7	0

### Transgenic expression of wild-type Fhod3 under the control of the β-MHC promoter rescues cardiac defects of Fhod3-null embryos

**Fig 2:**
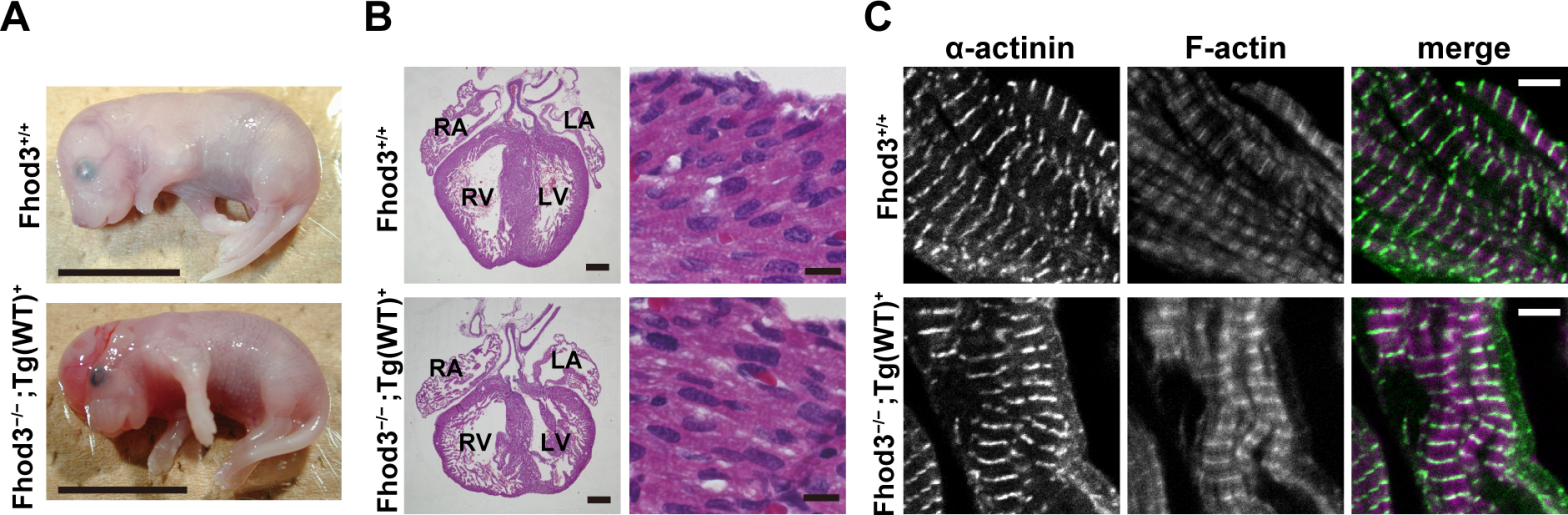
Rescue effect of transgenic expression of Fhod3 in the embryonic heart of *Fhod3*^−/−^ mice. (A) Whole mount analysis of wild-type (*Fhod3*^**+/+**^) and *Fhod3*^−/−Tg(β-MHC-Fhod3WT)^ (*Fhod3*^−/−^;*Tg(WT)*^**+**^) embryos at E18.5. Scale bars, 1 cm. (B) Histological analysis of wild-type (*Fhod3*^**+/+**^) and *Fhod3*^−/−Tg(β-MHC-Fhod3WT)^ (*Fhod3*^−/−^;*Tg(WT)*^**+**^) embryos at E18.5. LA, left atrium; RA, right atrium; LV, left ventricle; RV, right ventricle. Scale bars: (left) 300 μm; (right) 10 μm. (C) Confocal fluorescence micrographs of cardiac myofibrils of wild-type (*Fhod3*^**+/+**^) and *Fhod3*^−/−Tg(β-MHC-Fhod3WT)^ (*Fhod3*^−/−^;*Tg(WT)*^**+**^) embryos at E18.5. Sections of embryonic hearts were subjected to immunofluorescent staining for α-actinin (green) and phalloidin staining for F-actin (magenta). Scale bars, 5 μm.

In addition to the generation of transgenic mice expressing a mutant Fhod3 carrying the I1127A substitution, we also generated transgenic mice expressing a wild-type Fhod3 under the control of the β-MHC promoter (*Fhod3*^Tg(β-MHC-Fhod3WT)^). We obtained only one line expressing wild-type Fhod3, which appeared phenotypically normal and survived to adulthood. To investigate whether the wild-type Fhod3 protein expressed under the control of the β-MHC promoter is functional in the embryonic cardiogenesis, we performed a transgenic rescue experiment with *Fhod3*^*−/−*^ mice, which are known to be embryonic lethal [15]. Although intercrosses of *Fhod3*^*+/−*^ and *Fhod3*^+/−Tg(β-MHC-Fhod3WT)^ did not produce any *Fhod3*^−/−Tg(β-MHC-Fhod3WT)^ mice, analysis of embryos revealed that *Fhod3*^−/−Tg(β-MHC-Fhod3WT)^ embryos develop well beyond the *Fhod3*^*−/−*^ lethal stage and grow up to just before birth (Table 3). As shown in Fig 2A, *Fhod3*^−/−Tg(β-MHC-Fhod3WT)^ embryos at E18.5 were indistinguishable from wild-type embryos at the morphological level except for exencephaly, which was attributed to Fhod3 deficiency in the brain [15]. Histological analysis of embryos showed that *Fhod3*^−/−Tg(β-MHC-Fhod3WT)^ embryos have a well-developed heart composed of mature myofibrils (Fig 2B). Myofibrils of *Fhod3*^−/−Tg(β-MHC-Fhod3WT)^ embryos exhibited regularly spaced bands of sarcomeric α-actinin along with a striated F-actin pattern (Fig 2C). Thus, the wild-type Fhod3 protein expressed under the control of the β-MHC promoter in embryonic cardiomyocytes can function properly to achieve successful maturation of myofibrils. Taken together with the finding that *Fhod3*^+/+Tg(β-MHC-Fhod3WT)^ embryos also have a normally developed heart with fine myofibrils (S2 Fig), the exogenous wild-type Fhod3 protein does not affect heart development during the embryonic stage.

**Table 3:** Genotypes of embryos from mating of *Fhod3*^+/–^and *Fhod3*^+/–Tg(β-MHC-Fhod3WT)^.

Stage	*Fhod3*^+/+^	*Fhod3*^+/+^*;Tg(WT)*^+^	*Fhod3*^+/–^	*Fhod3*^+/−^*; Tg(WT)*^+^	*Fhod3*^–/–^	*Fhod3*^−/−^*;Tg(WT)*^+^
E9.5 –E10.5	3	2	3	1	2	2
E11.5 –E12.5	0	3	2	2	1	1
E13.5 –E15.5	3	4	9	13	0	4
E16.5 –E18.5	3	3	8	4	0	5

### Localization of expressed wild-type Fhod3 in the assembling myofibrils

**Fig 3:**
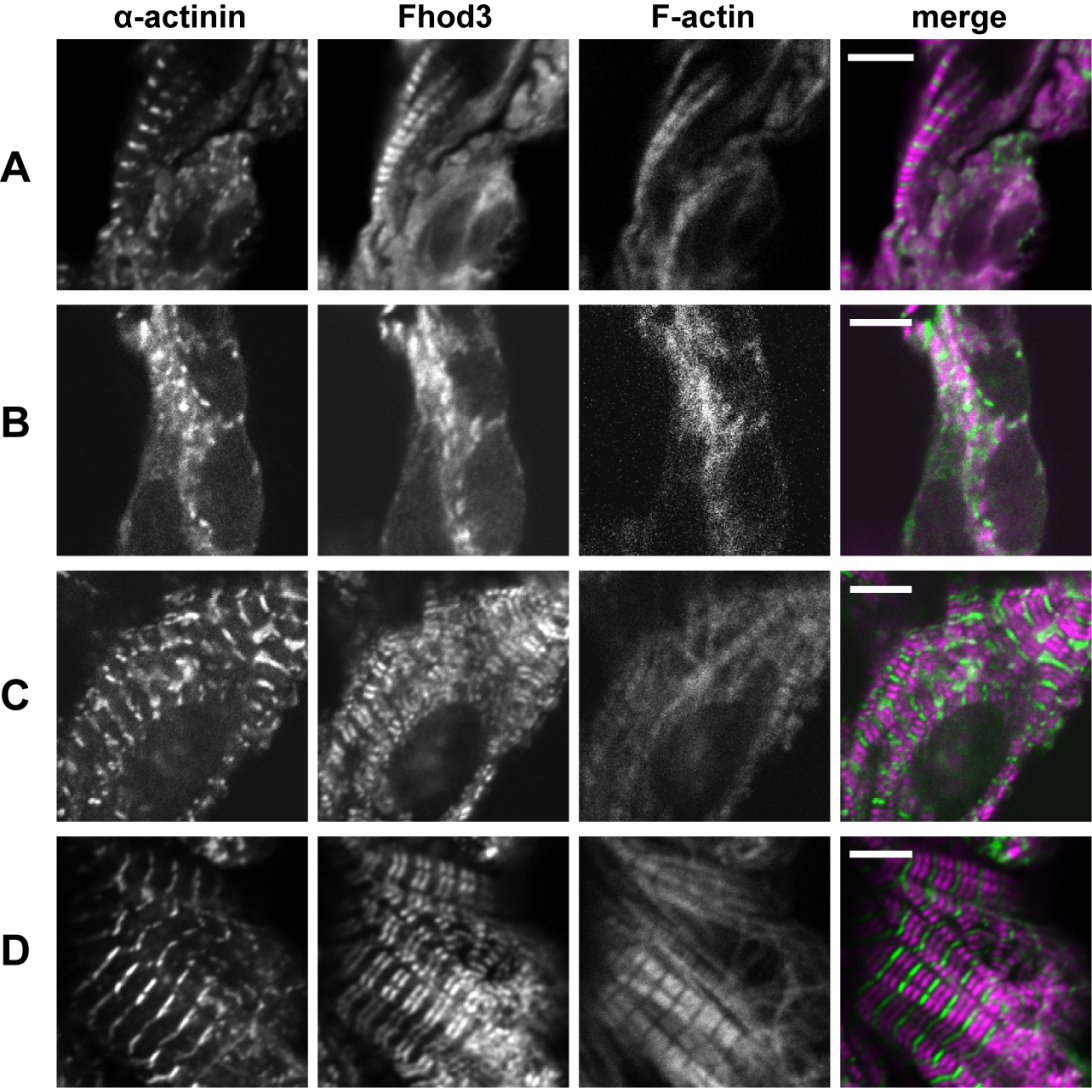
Sarcomeric localization of wild-type Fhod3 in the assembling myofibrils in the embryonic heart. Confocal fluorescence micrographs of cardiac myofibrils of *Fhod3*^Tg(β-MHC-Fhod3WT)^ embryos at E9.5 (A, B), E10.5 (C), or E13.5 (D). Sections of embryonic hearts were subjected to immunofluorescent staining for α-actinin (green) and Fhod3 (magenta) followed by phalloidin staining (not shown in merge). Scale bars, 5 μm.

We have previously demonstrated that Fhod3 null mice exhibit defects in cardiac myofibrillogenesis after E9.5, although premyofibrils were normally formed until E8.5 [15]. This observation indicates that the function of Fhod3 becomes critical after the formation of premyofibrils, *i*.*e*., during the maturation stage of myofibrils (E9.5–E11.5). Nevertheless, we could not determine the localization of Fhod3 during this stage probably due to the low expression level and limited sensitivity of Fhod3 antibodies [15]. To solve this problem, we examined Fhod3 localization using *Fhod3*^Tg(β-MHC-Fhod3)^ embryos; Fhod3 expression driven by the β-MHC promoter was markedly higher than that by the endogenous and the α-MHC promoters [19] in the embryonic heart (S1 Fig). As shown in Fig 3A, exogenously expressed Fhod3 was already found as two closely spaced bands between the α-actinin-containing Z-bands in developing myofibrils at E9.5. In less developed myofibrils at the same stage, Fhod3 localized in a punctate manner beneath the cell membrane; some dots of Fhod3 appeared to localize between the α-actinin-containing dot-like Z-bodies in an alternating pattern (Fig 3B). At E10.5, paired bands of Fhod3 located in the middle of the sarcomere (delimited by two Z bands) were observed throughout the cell body (Fig 3C). Subsequently, by E13.5, the distribution pattern of Fhod3 became fully maturated, which was almost identical to that observed in adult cardiomyocytes [19] (Fig 3D). These findings indicate that the Fhod3 already localizes to the specific region of the center of nascent sarcomeres preceding myofibril maturation, and continues to remain in the same central region throughout the myofibril maturation.

### Sarcomeric localization of Fhod3 precedes the completion of F-actin organization

**Fig 4:**
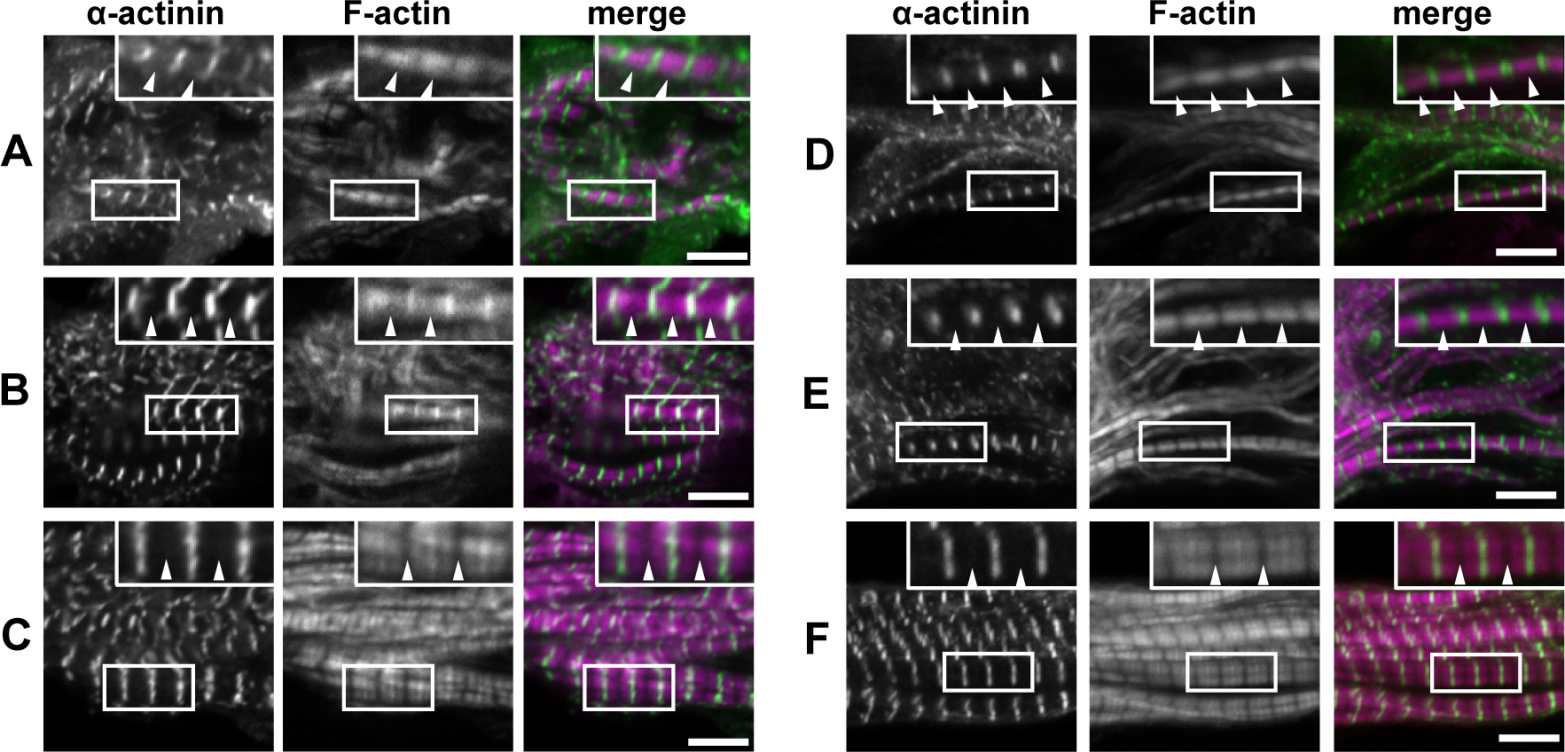
F-actin organization and α-actinin localization in the assembling myofibrils in the embryonic heart. Confocal fluorescence micrographs of cardiac myofibrils of *Fhod3*^Tg(β-MHC-Fhod3WT)^ embryos at E9.5 (A), E10.5 (B), or E13.5 (C), and those of wild-type (*Fhod3*^**+/+**^) embryos at E9.5 (D, F), or E13.5 (F). Sections of embryonic hearts were subjected to immunofluorescent staining for α-actinin (green) and phalloidin staining for F-actin (magenta). Arrowheads indicate the center of the sarcomere. Scale bars, 5 μm.

**Fig 5:**
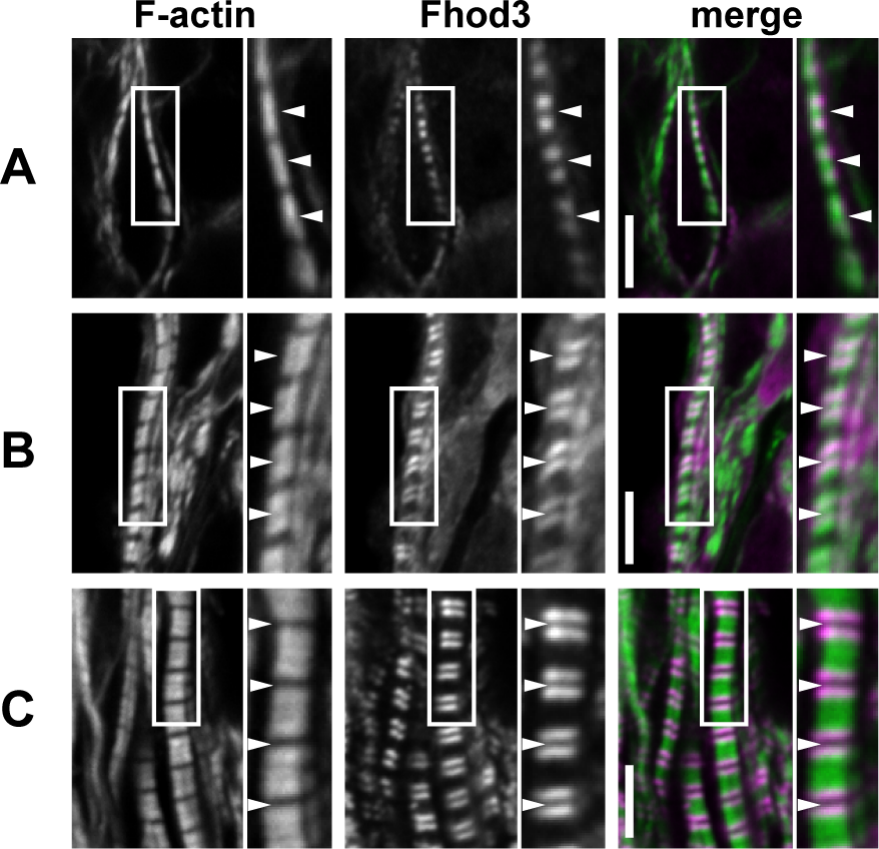
Fhod3 localization and F-actin organization in the assembling myofibrils in the embryonic heart. Confocal fluorescence micrographs of cardiac myofibrils of *Fhod3*^Tg(β-MHC-Fhod3WT)^ embryos at E9.5 (A), E10.5 (B), or E13.5 (C). Sections of embryonic hearts were subjected to immunofluorescent staining for Fhod3 (magenta) and phalloidin staining for F-actin (green). Arrowheads indicate the center of the sarcomere. Scale bars, 5 μm.

We further focused on the relationship between F-actin assembly and Fhod3 localization. During the process of myofibrillogenesis, actin filaments undergo dynamic rearrangement to form mature thin filaments of uniform length and polarity [3,24]. This maturation process of actin filaments can be estimated by phalloidin staining. At E9.5 as shown in Fig 4A, phalloidin exhibited a staining pattern that spans throughout the sarcomere except for the Z-line, where the accessibility of phalloidin appeared to be restricted [25]. It should be noted that no attenuation of phalloidin staining was observed at the central region of the sarcomere. At E10.5, myofibrils grew radially, *i*.*e*., the width of the Z-line became expanded and F-actin content was increased. Concomitantly, at a central region of the sarcomere, phalloidin staining became blurred, presumably reflecting that the pointed ends of actin filaments began to become aligned (Fig 4B). At E13.5, when myofibrillogenesis was almost completed, a clear gap in the middle of sarcomeres (*i*.*e*., the H-zone) had formed; actin filament lengths were restricted so that their pointed ends were aligned in line (Fig 4C). The time course of F-actin organization in *Fhod3*^Tg(β-MHC-Fhod3WT)^ embryos was essentially identical to that in C57BL/6 embryos (Fig 4D–4F). Intriguingly, during these processes of actin reorganization, the relative position between Fhod3 and phalloidin staining drastically changed (Fig 5), although that between Fhod3 and Z-bands was remained constant (Fig 3). As shown in Fig 5A, the central region of sarcomeres, *i*.*e*., a narrower gap of Fhod3 doublet bands was filled by F-actin at E9.5. At E10.5, phalloidin staining became weak at the narrower space of Fhod3 doublet bands (Fig 5B). At E13.5, a clear gap of phalloidin staining was formed at the narrow gap of Fhod3 doublet bands (Fig 5C). Thus Fhod3 localized at the central region of sarcomeres before the appearance of the H-zone, a region free of actin filaments, and H-zones formed between the Fhod3 gaps.

### Localization of Fhod3 with respect to sarcomeric components in assembling myofibrils

**Fig 6:**
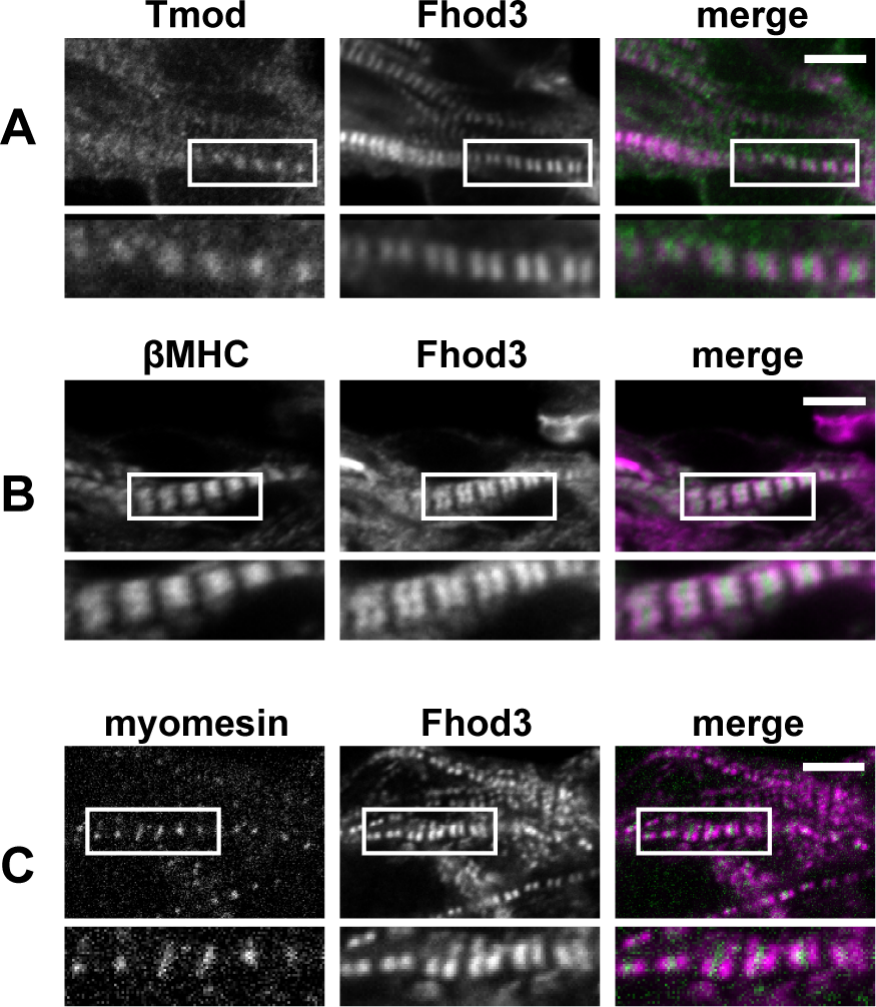
Localization of Fhod3 and other sarcomeric components in the assembling myofibrils in the embryonic heart. Confocal fluorescence micrographs of cardiac myofibrils of *Fhod3*^**Tg(β-MHC-Fhod3WT)**^ embryos at E9.5. Sections of embryonic hearts were subjected to immunofluorescent staining for Fhod3 (magenta) and Tmod (green) (A), β-MHC (green) (B), or myomesin (green) (C). Scale bars, 5 μm.

Finally we doubly stained embryonic heart sections for Fhod3 and other sarcomeric proteins to investigate the detailed localization of Fhod3 with respect to other sarcomeric proteins in the assembling myofibrils. Tropomodulin (Tmod), a pointed-end actin filament capping protein, is known to be involved in myofibril maturation through controlling the length of thin actin filaments at the center of the sarcomere [4]. As shown in 6A, Tmod localized as blurred dots between paired bands of Fhod3 at the center of the sarcomere, which is consistent with the finding that the pointed end of actin filaments is not yet aligned at E9.5. On the other hand, myosin heavy chain (MHC) localized as well-defined bands at the same stage (Fig 6B), indicating that the alignment of thick filaments precedes that of thin filaments, as described previously [26]. Myomesin, a component of the M-line [27], was located in a narrower gap of Fhod3 bands (Fig 6C), confirming the localization of Fhod3 at the center of the sarcomere during myofibrillogenesis.

## Discussion

In the present study we show that overexpression of a mutant Fhod3 (I1127A) protein, defective in an actin-assembling activity, during the embryonic stage disrupts myofibrillogenesis and results in mid-gestation lethality. The formin family proteins are known to function as a dimer [28,29]. Since the mutant Fhod3IA protein can interact with the wild-type Fhod3 protein (Fig 1D), the mutant Fhod3 protein is supposed to form a non-functional heterodimer with the endogenous wild-type Fhod3 protein, leading to a dominant negative effect. Thus the actin binding-activity of the FH2 domain of Fhod3 is critical for *de novo* myofibrillogenesis during embryonic cardiogenesis. The expression of Fhod3 is maintained thereafter throughout embryogenesis (S3 Fig) and after birth [19]. Taken together with our previous observation that overexpression of the mutant Fhod3 (I1127A) after birth causes the cardiomyopathic change in normally developed hearts [15], the actin binding-activity of the FH2 domain seems to be critical throughout life; both during *de novo* assembly and maintenance of myofibrils.

**Fig 7:**
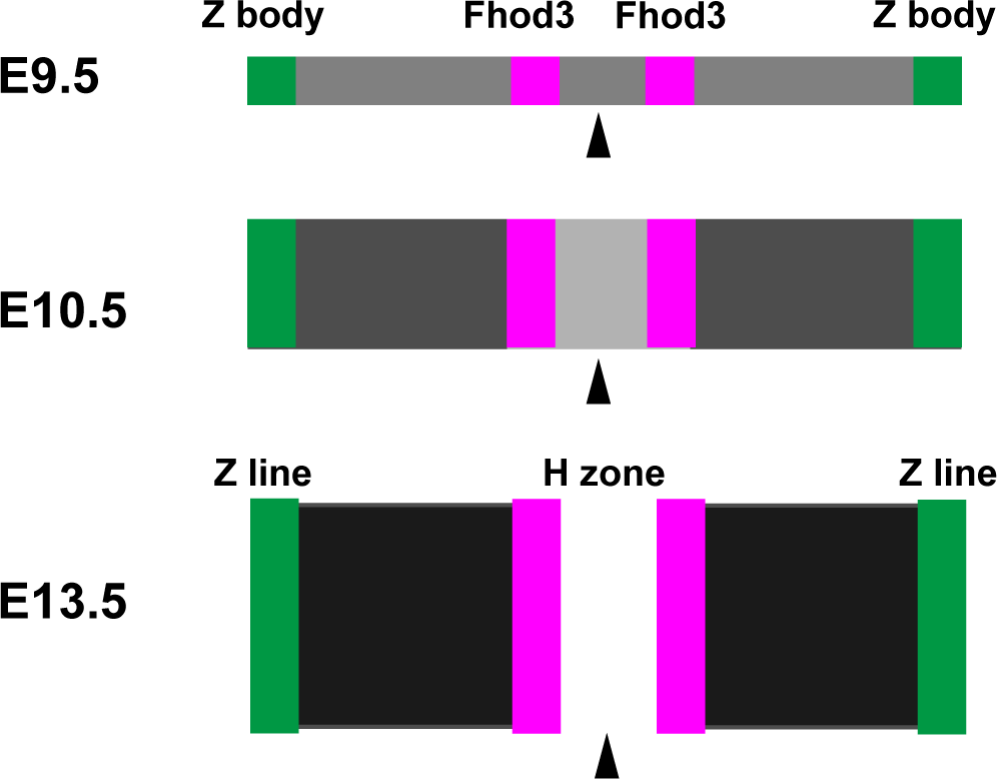
Schematic representation of the sarcomeric structure at embryonic stages. Relative localization of Fhod3 to α-actinin and F-actin shown in Figs 3–5 is schematically represented. F-actin content is represented in gray tones. Arrowheads indicate the center of the sarcomere.

We have also demonstrated that Fhod3 protein accumulates at the specific region of the center of sarcomeres at E9.5, a stage when myofibrillogenesis initiates. By contrast, Iskratsch *et al*. have recently shown that Fhod3 is diffusely localized at E9.5 [18], which appears to be inconsistent with our observations. However, some intense signals of Fhod3 in their report seemed be distributed in an alternating pattern with troponin T and in an overlapping pattern with myosin (see Figs 2 and 4 in Ref. [18]); these patterns are in agreement with our present result that Fhod3 is localized in the central sarcomere. Thus Fhod3 likely localizes at the center rather than the boundary of sarcomeres during the early stage of myofibrillogenesis. The localization pattern is maintained until adulthood in our previous report [15,19], but not in the reports by Iskratsch et al [14,18]. The reason for this discrepancy is presently unknown, although it might be related to differences in antibodies and fixation protocols as discussed previously [19]. At least in our hands, Fhod3 accumulates at the center of sarcomeres throughout myofibrillogenesis (Figs 3–5; schematically represented in Fig 7). During the process of myofibril maturation, F-actin content is progressively increased, which appears to be achieved by Fhod3 [15]. At the same time, thin actin filament lengths are restricted so that their pointed ends are aligned, forming a clear gap of phalloidin staining in the central region of sarcomeres (*i*.*e*., the H-zone). Since the striated pattern of actin filaments (*i*.*e*., emergence of H-zones) was not observed in Fhod3 null embryos [15], Fhod3 was also expected to contribute to this process. Our present findings that Fhod3 accumulates at the central region before the emergence of H-zones and that H-zones are observed after Fhod3 accumulation (Figs 5 and 7) suggest that Fhod3 promotes these reorganizing processes of thin actin filaments at the middle of the sarcomere.

Fhod3 thus localizes mainly to the center of the sarcomere but rarely to the Z-line where the barbed ends of thin actin filaments are anchored, albeit the FH2 domain of Fhod3 interacts with the barbed end of actin filaments in a previous *in vitro* assay [13]. This seemingly paradoxical localization of Fhod3 can be explained by our hypothesis that formins mediate end-to-end annealing to the pointed end of thin filaments [13]. If short filaments capped at their barbed ends by Fhod3 are incorporated to the pointed end of thin filaments through end-to-end annealing, Fhod3 might be present around the middle of the sarcomere. Recently, Molnár *et al*. have reported that the *Drosophila* formin DAAM also localizes at the central region of sarcomeres in developing larval body wall muscles [30]. Since dDAAM seems to have an end-to-end annealing activity, our hypothesis may be applicable to other sarcomeric formins. On the other hand, the localization period seems to be different between dDAAM and Fhod3; dDAAM localization at the center of sarcomeres is decreased during development, whereas Fhod3 localization seems to be retained throughout life from a very early stage of the embryo (this study) up to adulthood [19]. Since the cardiac muscle constantly beats throughout life, sarcomeres in cardiomyocytes are supposed to be damaged constantly from by myosin-driven force at the central region. Therefore, localized reorganization of actin filaments might occur constantly at the central region in cardiac muscles unlike other striated muscles. Through the end-to-end annealing to the pointed end of thin filaments, Fhod3 might mediate reorganization of actin filaments not only for myofibrillogenesis but also for the maintenance of sarcomeres. On the other hands, it is possible that Fhod3 localization to the center of the sarcomere is mediated not by the FH2-mediated association with actin filaments but by interactions with other sarcomeric proteins. Indeed, Fhod3S, a short splice variant that lacks 151 amino acids in the N-terminal region, shows weak localization to the center of the sarcomere [13], suggesting the possibility that the N-terminal region is involved in the localization of Fhod3, although target proteins have not been identified. Future studies are awaited to elucidate the molecular mechanism for Fhod3 localization and function.

## Supporting Information

S1 Fig Cardiac expression of Fhod3 in the transgenic embryos expressing Fhod3. Cardiac tissue lysates from embryos of the indicated genotypes at E9.5 (left panels) and at E18.5 (right panels) were analyzed by immunoblot with the anti-Fhod3-(C-20) antibodies.
(TIF)

S2 Fig Effect of transgenic expression of Fhod3 in the embryonic heart of *Fhod3*^+/+^ mice. (A) Whole mount analysis of *Fhod3*^+/+Tg(β-MHC-Fhod3WT)^ embryos at E17.5. Scale bars, 1 cm. (B) Histological analysis of *Fhod3*^+/+Tg(β-MHC-Fhod3WT)^ embryos at E17.5. LA, left atrium; LV, left ventricle; RV, right ventricle. Scale bars: (left) 300 μm; (right) 10 μm. (C) Confocal fluorescence micrographs of cardiac myofibrils of *Fhod3*^+/+Tg(β-MHC-Fhod3WT)^ embryos at E17.5. Sections of embryonic hearts were subjected to immunofluorescent staining for α-actinin (green) and phalloidin staining for F-actin (magenta). Scale bars, 5 μm.
(TIF)

S3 Fig Cardiac expression of endogenous Fhod3 in embryos and neonates. Cardiac tissue lysates from C57BL/6 mice at the indicated days were analyzed by immunoblot with the anti-Fhod3-(C-20) antibodies.
(TIF)
