# Supplementary figures and images for: Transgenic Expression of the Formin Protein Fhod3 Selectively in the Embryonic Heart: Role of Actin-Binding Activity of Fhod3 and Its Sarcomeric Localization during Myofibrillogenesis
Source: PLoS One. 2016 Feb 5;11(2):e0148472. doi: 10.1371/journal.pone.0148472 (PMC4744011; doi:10.1371/journal.pone.0148472)

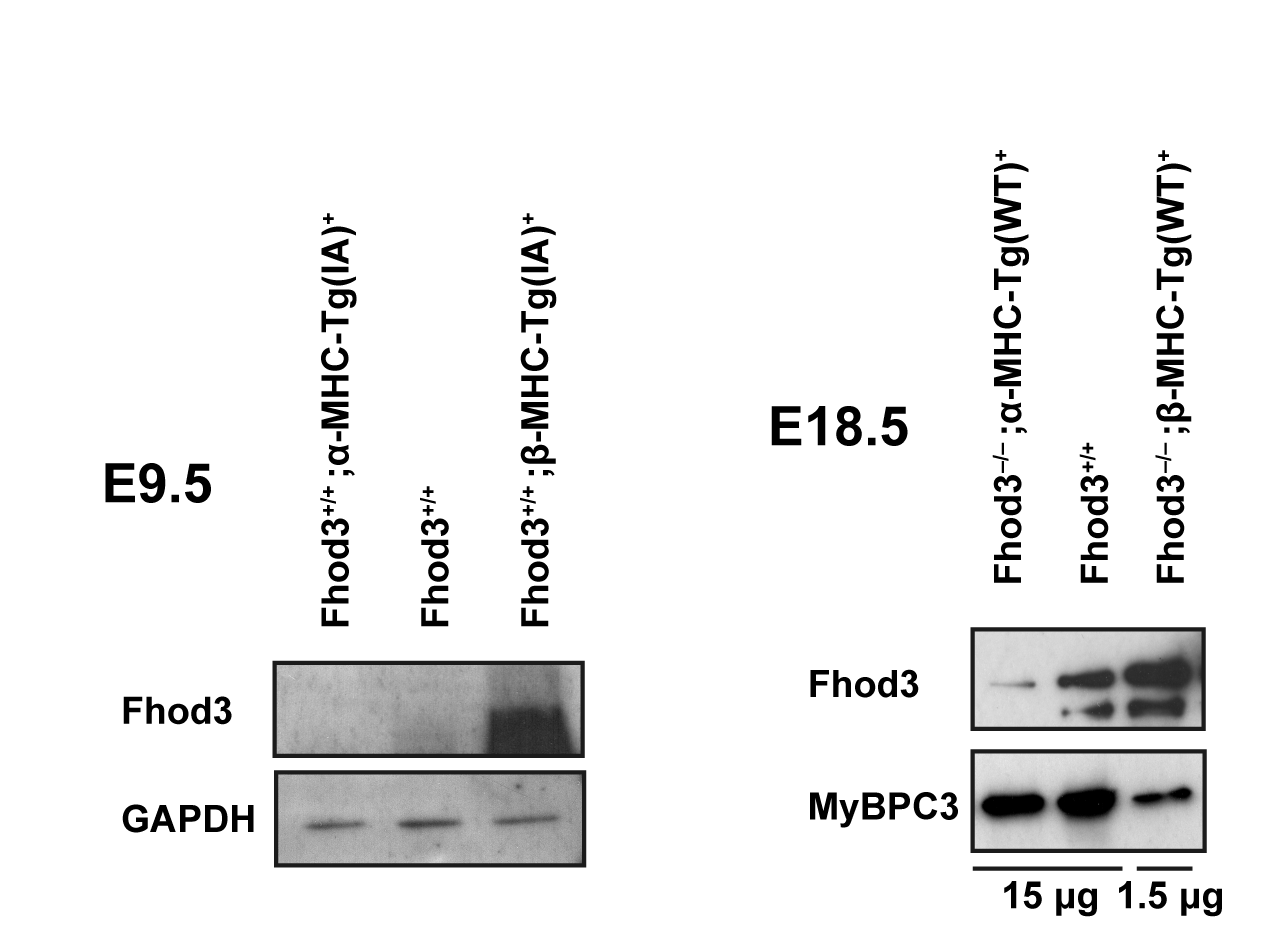

Supplement: S1 Fig — Cardiac tissue lysates from embryos of the indicated genotypes at E9.5 (left panels) and at E18.5 (right panels) were analyzed by immunoblot with the anti-Fhod3-(C-20) antibodies. (TIF) [file pone.0148472.s001.tif]

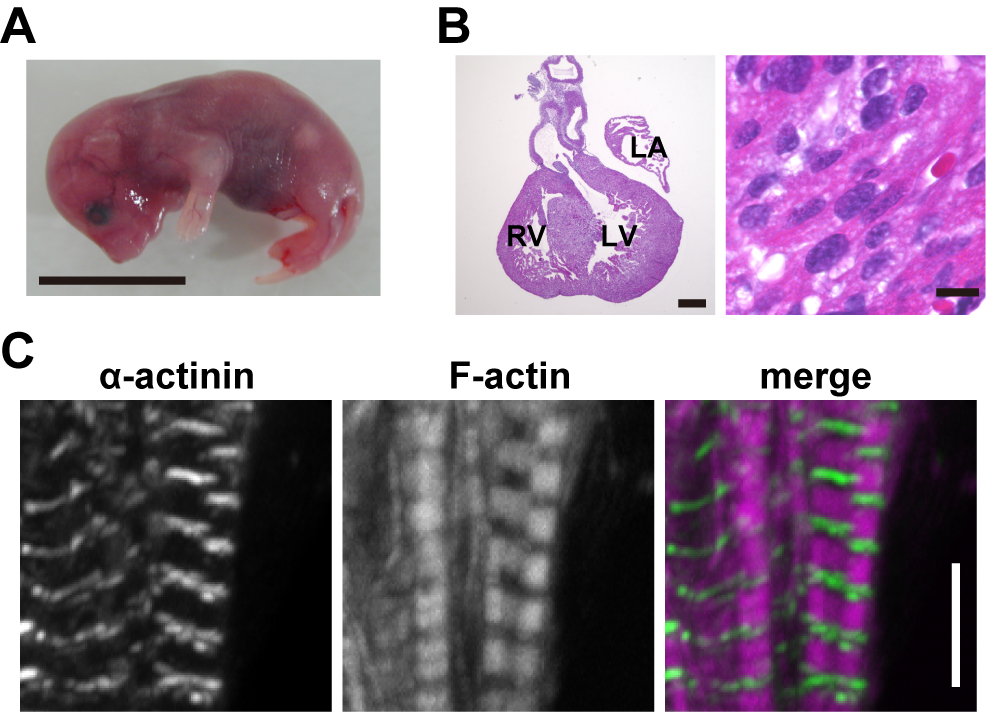

Supplement: S2 Fig — (A) Whole mount analysis of Fhod3+/+Tg(β-MHC-Fhod3WT) embryos at E17.5. Scale bars, 1 cm. (B) Histological analysis of Fhod3+/+Tg(β-MHC-Fhod3WT) embryos at E17.5. LA, left atrium; LV, left ventricle; RV, right ventricle. Scale bars: (left) 300 μm; (right) 10 μm. (C) Confocal fluorescence micrographs of cardiac myofibrils of Fhod3+/+Tg(β-MHC-Fhod3WT) embryos at E17.5. Sections of embryonic hearts were subjected to immunofluorescent staining for α-actinin (green) and phalloidin staining for F-actin (magenta). Scale bars, 5 μm. (TIF) [file pone.0148472.s002.tif]

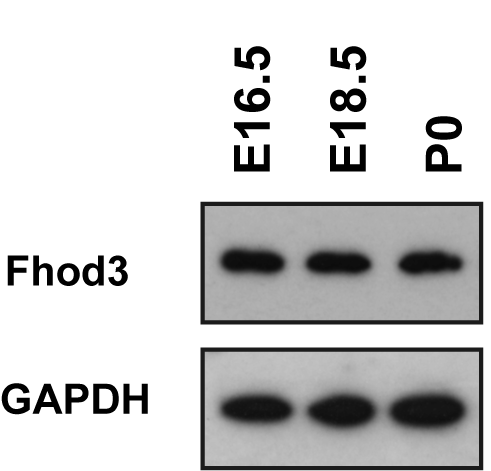

Supplement: S3 Fig — Cardiac tissue lysates from C57BL/6 mice at the indicated days were analyzed by immunoblot with the anti-Fhod3-(C-20) antibodies. (TIF) [file pone.0148472.s003.tif]
